# “Ah, it's best not to mention that here:” Experiences of LGBTQ+ health professionals in (heteronormative) workplaces in Canada

**DOI:** 10.3389/fsoc.2023.1138628

**Published:** 2023-04-03

**Authors:** Stephanie R. Bizzeth, Brenda L. Beagan

**Affiliations:** ^1^Community Mental Health and Addictions, Nova Scotia Health Authority, Dartmouth General Hospital, Dartmouth, NS, Canada; ^2^School of Occupational Therapy, Dalhousie University, Halifax, NS, Canada

**Keywords:** Canada, health professionals, heterosexism, LGBTQ+, minority group, queer

## Abstract

**Introduction:**

Despite human rights protections for lesbian, gay, bisexual, transgender, and queer (LGBTQ+) people, LGBTQ+ professionals may continue to experience discrimination working in heteronormative systems and spaces.

**Methods:**

In this qualitative study 13 health professionals (nurses, occupational therapists, and physicians) from across Canada participated in in-depth qualitative interviews to explore their experiences with work-related microaggressions and heteronormativity.

**Results:**

Heterosexist microaggressions from both patients/clients and colleagues were the norm, perpetuating and bolstered by heteronormative workplace and professional cultures. In turn, LGBTQ+ professionals navigated disclosure-decision-making, in power-laden contexts where all options carried potential negative consequences.

**Discussion:**

Drawing on the notion of “heteroprofessionalism,” we argue that the concept of professional carries encoded within it demands that the occupant of that category be—or present as—heterosexual, an unmarked status that can be readily desexualized. Acknowledging sex and sexuality disrupts “professionalism.” We argue that such disruption, indeed dissention, is necessary to open (hetero)professional spaces to LGBTQ+ workers.

## Introduction

In general, discussions within the clinic occurred under the assumption that all persons in the clinic view heterosexuality as normal, acceptable, and worth celebrating through sharing… There was a certain ease that came from an expected appreciation and understanding of the topic (Jackson, 2000, p. 30).

Almost 25 years ago, Jeanne Jackson documented the experiences of lesbian occupational therapists, noting that subtle exclusion meant the health professionals in her study missed out on informal social connections through which much practice knowledge was articulated and solidified. Participants highlighted informal lunch table chit-chat as rife with assumptions and expectations of heterosexuality, intermeshed with important information-sharing and patient/client problem-solving. Lesbian therapists were excluded or absented themselves due to discomfort; in either case they missed out on a critical component of workplace camaraderie, mutual support, and co-learning.

Jackson does not use the language of heteronormativity, which was still very new at that time, but that is precisely what her analysis describes. Based on the earlier concept of “compulsory heterosexuality” (Rich, 1980), heteronormativity is an ideological stance in which heterosexuality is both assumed—understood as normal, natural, inherent—and prescribed—understood as the only “right” way to be, the way people *should* be (van der Toorn et al., 2020). Heteronormativity renders non-heterosexual identities overlooked, dismissed and devalued as inferior or deviant. It is fortified and legitimated by heterosexism, the oppression of those who live, love and identify outside the bounds of heterosexual norms, ranging from dominance in social institutions like media, politics and education, to violence and the threat of violence. Heteronormativity intertwines with cisnormativity, the insistence that gender is binary, with gender identity and expression inextricably mapped onto (presumed binary) biological sex (Brady et al., 2022). Cisnormativity privileges those whose gender identity aligns with the gender they were assigned at birth (cisgender).

While the heteronormativity of health professional cultures may well have lessened in the quarter century since Jackson wrote, there is evidence suggesting this may not be the case, despite substantial improvements in human rights protections in most places (Eliason et al., 2018; Toman, 2019; Turban, 2019; Cleland and Razack, 2021). Despite changing attitudes and improved legal protections, lesbian, gay, bisexual, transgender and queer (LGBTQ+) people continue to confront the stranglehold of heteronormative workplace environments (Eliason et al., 2018; Resnick and Paz Galupo, 2019; van der Toorn et al., 2020; Worthen, 2021). LGBTQ+ workers endure routine messaging from managers, colleagues and workplace cultures that indicate less than full belonging (Nadal, 2019).

In this article we explore the experiences of 13 self-identified LGBTQ+ health professionals across Canada. Our main objective is to examine how heterosexist microaggressions and institutionalized heteronormativity shape their everyday work experiences, harming them and constraining their engagement in their professional work. We explore how available responses to microaggressions and heteronormativity prove not only insufficient, but also contribute to the continued heteronormativity of professional work contexts.

## Heteronormativity and microaggressions in the professions

In the context of expanding human rights protections, one of the key ways heteronormativity is policed and enforced in workplaces and professional cultures is through microaggressions: disparaging comments, jokes, avoidant behaviors, being overlooked or discounted, being tokenised or exoticized. Microaggressions may be defined as the “brief and commonplace daily verbal, behavioral, or environmental indignities, whether intentional or unintentional, that communicate hostile, derogatory, or negative… slights and insults toward members of oppressed groups” (Nadal, 2008, p. 23). They can be interpersonal or environmental, built into institutional policies and practices. Microaggressions at work may lead to anxiety, suspicion and distrust, depression, withdrawal, ostracism and/or isolation, doubt about one's value in the workplace and other psychological and physiological distress (Eliason et al., 2018; Gabrani and Pal, 2019; Resnick and Paz Galupo, 2019; Vaccaro and Koob, 2019; Bullock et al., 2021). Heterosexist microaggressions regulate and control workers through fear—fear of interpersonal hostility or harm, fear of institutional punishment and fear of isolation (Mizzi, 2013). These are no less relevant in the professions than in other workplaces.

Within discourses of “professionalism,” some ways of being and doing, some subjectivities and some bodies are deemed acceptable and appropriate while others are disavowed. It has been argued that professionalism is structured by a politics of respectability, which demands that members of socially marginalized groups regulate their bodies and self-presentations to adhere to normative standards, if they want acceptance and the privileges of membership (Davies and Neustifter, 2021; Beagan et al., 2022). LGBTQ+ embodiments have the potential to disrupt respectability, rendering them incommensurable with professionalism (Davies and Neustifter, 2021). LGBTQ+ workers, then, are expected to manage and control those aspects of their identities that are controversial, disruptive, those that “do not conform to dominant norms for professionalized self-presentation” (Davies and Neustifter, 2021, p. 6). Mizzi coined the term “heteroprofessionalism” to capture this “demand for a standardized professional identity void of same-sex desire” which in turn relegates LGBTQ+ identity to a “silenced aspect of the self” (Mizzi, 2013, p. 1,620–21). Sex and sexuality are considered outside the bounds of professionalism, desexualizing all workers, and pushing those whose social identities are defined through sexuality—LGBTQ+ workers—to the margins (Mizzi, 2013; Calvard et al., 2020). Even within workplaces that self-proclaim inclusion for LGBTQ+ people, heteroprofessionalism withholds full acceptance contingent upon approximating heteronormative expectations. Those who most resist normalizing forces, disrupting through their very existence, may find their professional recognition tenuous indeed.

Stereotypes of LGBTQ+ bodies and lives may limit professionals' control over the degree to which they reveal or conceal LGBTQ+ identities. They may be read as LGBTQ+ against their will, or they may be read as cis-heterosexual, despite identity disclosures (Einarsdóttir et al., 2016). The prevalence of narrow stereotypes means they may face negative consequences both for failing to embody and perform heterosexuality, and for failing to embody and perform queerness as it has been constructed by coworkers and others in their workplace hierarchies. In other words, they may be punished for being too queer, not queer enough, or not the right kind of queer (Einarsdóttir et al., 2016; Stenger and Roulet, 2018).

In the context of heteroprofessionalism, policed by microaggressions and regulated by fear of consequences, some LGBTQ+ professionals may choose to “pass” (Goffman, 1963), hiding sexual and gender identities from clients and/or colleagues to avoid stigma and to assimilate into existing power structures. Some may choose to “cover” (Goffman, 1963) queerness, downplaying its significance to decrease stigma and potential harms. Yoshino (2006) suggests covering occurs on four axes: appearance (dress, grooming, and bodies), affiliation (alignment with LGBTQ+ cultures), activism (politicization), and association (social networks, partners, and identity-specific groups). Covering, or toning down queerness to make it less objectionable, can improve chances of infiltrating existing power structures. Thus, as Yoshino notes, covering may be an individually chosen identity-management strategy, but it may also be subtly coerced, with institutions and professional cultures offering inclusion at the price of (near) assimilation. Covering is both predicated on and simultaneously supports “respectability hierarchies,” allowing those who can assimilate or approach outward conformity to achieve inclusion and social respectability at the expense of others (Branfman, 2015, p. 73). It is critical to remember that such respectability hierarchies are rooted in and operationalized through heteronormative workplace cultures.

## Health professional contexts

Within the health professions, heteronormativity is entrenched, expressed and taught through formal and informal curricula, and through professional cultures of conformity (Jackson, 2000; Risdon et al., 2000; Eliason et al., 2011a,b, 2018; Röndahl, 2011; Robertson, 2017; Murphy, 2019; Turcotte and Holmes, 2021). “Professional behavior” is assessed and evaluated, with discourses of professionalism masking the demands that new entrants comply with expectations of bodies, comportment and behavior that are inherently white, Western, middle-to-upper-class, heterosexual and cis-masculine (Beagan, 2000, 2001; Martimianakis et al., 2009; Jenkins et al., 2021). Particular ways of being are deemed “correct,” subject to both formal and informal surveillance (e.g., MacKenzie and Merritt, 2016), with “unprofessional” behaviors cause for “remedial” action. As Mizzi has argued, “Professionalism can be an instrument of inequity and injustice by victimizing and punishing victims of discrimination to a point that it establishes a culture of fear for individuals with non-normative identities” (Mizzi, 2013, p. 1,604). In these contexts, LGBTQ+ health professionals and trainees—faced with pervasive heteronormativity and microaggressions—may opt to mask, hide or diminish the significance of their LGBTQ+ identities (Ross et al., 2022). This is intensified by the clear power hierarchies within and across health professions, leaving trainees and workers subject to the evaluation of powerful others. As one participant said, in a study with transgender and gender expansive (e.g., non-binary, genderqueer, and agender) physicians,

I found it very difficult to weigh my what I felt to be my duty to speak up against injustice with my desire to remain safe… there's a lot of power differential… I was very much afraid that not only would there be professional repercussions, but also there might be personal repercussions… (Westafer et al., 2022, p. 1).

Tracking change over time, Eliason et al. (2018) indicate that while experiences of overt harassment and ostracism may be declining over the past three decades, LGBTQ+ health professionals still routinely hear disparaging and stereotyping remarks about LGBTQ+ people, from both colleagues and patients/clients, and may witness ill-treatment of LGBTQ+ patients/clients and their family members, which simultaneously conveys contempt for their own identities. In a recent survey of medical graduate trainees (*n* = 730) LGBTQ+ respondents were significantly more likely to have experienced discrimination and microaggressions (Walker et al., 2022). Based on their qualitative research, Bullock et al. add that, “Patients, providers, peers, and the learning environment itself are all common sources of microaggressions” (Bullock et al., 2021, p. S71). LGBTQ+ healthcare workers experience microaggressions ranging from patients refusing to be seen by them (Eliason et al., 2018), to colleagues making inappropriate comments about their sexual/gender identities, ostensibly as jokes (Eliason et al., 2011a). It may be particularly hard to respond to microaggressions coming from patients/clients, given the demands of altruism and selfless sacrifice embedded in conceptualizations of professionalism (Gabrani and Pal, 2019; Turban, 2019; Sibbald and Beagan, 2022). LGBTQ+ health professionals in response must devote untold energy to navigating whether/when/how/to whom they disclose their identities.

In this critical interpretive qualitative study we explore the experiences of 13 self-identified LGBTQ+ healthcare workers from across three professions (medicine, nursing and occupational therapy) in Canada. We examine their day-to-day experiences in varied work environments with clients/patients and colleagues, their navigation of microaggressions and heteronormative professional climates, and their responses to heteronormativity, walking the disclosure/non-disclosure tightrope erected through heteroprofessionalism.

## Research methods

After obtaining research ethics approval from three universities, participants were recruited from across Canada using snowball sampling, social media and recruitment posters. Inclusion criteria were self-identification as LGBTQ+ and 5+ years of professional practice. Those who expressed interest were emailed study details and consent forms; once eligibility was confirmed, interviews were scheduled. The sample for this analysis included six occupational therapists, five physicians, and two nurses. Participants were given a $100 e-gift card in appreciation for their time and expertise.

Individual, semi-structured interviews were conducted by phone or in person, after discussing consent. Interviews averaged 60–90 min, exploring experiences of belonging and marginality in professional contexts, both during education and in workplaces Interviews were recorded, transcribed, deidentified and checked for accuracy. ATLAS.ti qualitative data analysis software was used to facilitate coding and inductive analysis of transcripts by a team. Some codes drew from theory and literature, while others were identified through reading and rereading the transcripts. In a reflexive approach to thematic analysis (Braun and Clarke, 2019), we moved iteratively between coded data and full transcripts, between theory and data, and among transcripts. For readability, quotations used in the manuscript were “cleaned” by removing false starts and filler words like “um” and “ah.”

Weekly team meetings over many months focused on data interpretation; collectively we pondered how we were thinking about codes, whether we needed new codes and how codes might be altered for greater nuance or accuracy (Braun and Clarke, 2021). Gradually our discussions engaged more with theory and other literature. While our understanding of heteronormativity and heterosexism as forms of oppression predated this analysis, the specific framework of heterosexist microaggressions and cultures of heteroprofessionalism was identified through ongoing team analysis and discussions, proving a useful structure for this article.

The research team included LGBTQ+ and heterosexual team members; all identified as cisgender, though our gender presentations vary. In every aspect of the research we strived not to eliminate biases, but rather to mobilize our lived experiences, our socially located perceptions and perspectives to enrich analyses. We employed a form of “transpersonal reflexivity” (Dörfler and Stierand, 2021), with perceptions, experiences and beliefs becoming sources of interpretive insight through collectively thinking aloud about the data.

## Results

Participants were mostly in their 30's, with some in their 40 and 50's. Most had been in practice 5–9 years, primarily in urban contexts. Of the 13 participants, four people explicitly identified as men, seven as women. Almost all were white and did not identify as disabled. In this article we analyze the experiences of participants under three main themes: Interpersonal microaggressions, heteronormativity in professional cultures, and responding to heteronormativity. In an effort to maintain confidentiality we do not identify quotations by ID#, or by demographics (e.g., age, practice area, specific gender or sexual identity, or other intersecting identities).

## Interpersonal microaggressions

For many participants, heterosexist microaggressions in professional settings were seen as the norm, a routine part of encounters with both patients/clients and colleagues. They required participants to navigate decisions about identity disclosure, calculating when safety trumped living their LGBTQ+ identities openly.

### Microaggressions from clients/patients

A few participants reported that blatant heterosexist aggressions from patients/clients were fairly routine, even normalized: “In my field we are used to patients sometimes making comments that are really quite unpleasant.” Some described it in ways that (implicitly or explicitly) characterized the hostility as a symptom, or a consequence of the stress surrounding acute illness:

I have had cases where families have made homophobic comments and things like that, or sometimes when working with really sick patients in the emerg who, you know they are acutely unwell or they have personality disorders, they might make really disparaging homophobic comments toward me.

More typical than such overt hostility were indirect and subtle experiences with clients/patients. For example, some participants described overhearing conversations clients had with others using derogatory heterosexist insults. They worried about the harmful impact of such verbal hostility on other people in the clinical setting who might overhear. Occasionally patients made generalized heterosexist comments directly to participants, not realizing the participants identified as LGBTQ+: “They'll just make some sort of comment, sort of making conversation with me, but not even realizing that it might, their opinion might actually be impacting me personally.”

It was particularly challenging to figure out how to respond to heterosexist microaggressions from patients/clients, given the relative power position of the health care professional. A common strategy was to ignore it and continue the clinical encounter: “If it's with a patient interaction, I have to say I try not to take it personally and I just do what I think would be most helpful for the patient and what I think is clinically relevant.” Responding “personally” tended to be characterized as “unprofessional.” One participant went further to suggest that confronting heterosexist microaggressions might be riskier for a LGBTQ+ health professional than for a cisgender heterosexual colleague:

I had been working with staff when I was a resident, and the patients would, you know, make some kind of derogatory comment or something and the staff really called them out on it and labeled it as inappropriate. And it made me wonder if maybe I let it go, vs. whereas maybe other people might have more of an issue with it and maybe call it out.

When not overtly disclosing LGBTQ+ identity, confronting heterosexist microaggressions risks eliciting “guilt by association,” potentially incurring stigma. This participant commented later that while working for change is important, “if the people in power don't like that, then they can kind of um, maybe make you in a position where you are more likely to be ostracized.”

### Microaggressions from colleagues

In their professional workplaces, participants noted that colleagues also employed language, comments and behavior that constituted heterosexist microaggressions. For example, one participant described routinely fielding questions from coworkers that mobilized and bolstered stereotypes of LGBTQ+ relationships:

In my first job, I'd say it was a different kind of homophobic, whatever. More ignorance… It was just, the questions that some of my coworkers would ask me, like, it felt like 1972. Like, “Which one of you in the relationship is the man?” I'm like, “I don't– Are you kidding me?!… How is it you're thinking that this is appropriate?”

One participant reported being bullied by a boss, which he perceived was due to his sexual identity. Another participant was highly uncomfortable when a manager mocked his voice and mannerisms, insinuating gay “flamboyance” was comedic.

At the same time, narrow stereotypes could be activated to demand particular forms of LGBTQ+ embodiment and performance. One gay man reported coworkers expected him to perform a specific version of “gayness” to fit their expectations: “How do I explain? I don't know. I'm definitely viewed as [Name] the Gay Guy. It's not actually said, but I think it's because… I fit the stereotypes in many ways… I find that's just expected now, almost.” The prescribed identity display felt obligatory.

Some participants felt tokenized, reduced to their queerness, with workplace colleagues employing nicknames like “Team Rainbow” that focused on their sexual identities. This focus, or even unwanted “outing,” could be uncomfortable, even when intended light-heartedly:

In my medical school, I kind of tried to foster some people being more comfortable talking about these things so we had a little group of us that kind of hung out, like 5 or 6 of us, and we kind of got labeled as the “Gay's Anatomy” of the medical school class. Which is in some ways funny but also not funny.

Participants reported being called on to interpret or represent queerness, an aspect of tokenism that assumes particular aspects of identity hold primacy:

In orientation week, cause I lived in a house with two medical students [and] we had offered to host an event where people go from house to house. And they decided to make us the “LGBTQ welcoming house” … It wasn't our idea. We had just signed up to host one of the houses in this welcoming event for the new med students and we kind of got labeled.

Such labeling makes sexual identity the key feature of someone's personhood—but only for LGBTQ+ people. Dominant sexual and gender identities remain unmarked.

In work contexts, participants found colleagues assumed all LGBTQ+ people knew or could readily identify each other.

I can't count the number of times… I will have people come up to me, like other physicians… allied health staff, and they're like, “Oh hey, that new med student, that new nurse, do you think they're gay?” … And I just look back at them and say, “Does it matter?” “Cause that is one part of a person's life, I don't see how it's relevant.”

The assumption that others can tell who is LGBTQ+ again mobilizes stereotypes of LGBTQ+ bodies and self-presentations, while simultaneously Othering LGBTQ+ co-workers (Einarsdóttir et al., 2016).

## Heteronormativity in professional cultures

Microaggressions targeting LGBTQ+ workers are both a product of and serve to support heteronormativity and heterosexism. Participants described their professional cultures as infused with heteronormative assumptions that contributed to LGBTQ+ invisibility and marginalization. They spoke about routine assumptions made by colleagues and clients/patients, and the ways questions and casual conversations conveyed powerful messages of not-fully-belonging.

Repeatedly, participants described the ways everyday interactions that are part of building rapport with patients were infused with heterosexist assumptions that excluded them, or left them suddenly scrambling to avoid or disarm a potentially volatile situation.

You're engaged in sort of chat, about whatever. And people would be asking me “Oh, are you married? Do you have kids?” And at the time, I wasn't married; I was single. “No, I don't have children.” And the look on their face, like they couldn't believe that.

It's always been just the same question: do you have a boyfriend; do you have a husband; do you have kids? Those three basic questions.

These are not ill-intended questions, conveying hostility, they simply assume—and by assuming impose as normative—heterosexuality.

I guess it's the presumption that's out there, that my life would be like everyone else's. So, for example, clients or coworkers who maybe don't know, the comments like, “Well, do you have children?” … So yeah, that presumption, I find that that has been frustrating. And then how do you, do you respond in a disclosing kind of way, or do you just ignore, or do you sort of?

Participants felt pressured to make complex disclosure decisions in the face of such heteronormative questions and assumptions. Occasionally, when participants felt safe enough to always be “out” at work, they found this enhanced connection with LGBTQ+ patients, facilitating common ground and stronger rapport.

With colleagues, heterosexist assumptions left participants caught between invisibility and unwanted hyper-visibility. For example, one participant described feeling Othered when in casual conversations with professional colleagues, never fitting their expectations but fearing talking about her life would label her as deviant:

You know, you go have a cup of tea or something with them, and it's like, you know, some of the questions: “Okay, well, where do you work? Where do you live? What's your family? Do you have a family? Do you have a husband?” Right? It's always that sort of, “Do you have a husband?” normative questions, always.

Some reported that when colleagues knew their LGBTQ+ identities, it seemed to stifle the usual co-worker chat that can lubricate workplace interactions: “We had a good working relationship, but they never asked me about my personal life, as they would the other colleagues… It's like, just missing out on some of those social conversations.”

Participants suggested that heteronormativity pervaded even the core content taught in their professions. For example, cis-heterosexual nuclear families were often presented as normative and universal in health professional education:

[In school] we were being taught to make assumptions about our patients... It was always “mom and dad.” And even if someone was going to try to be inclusive, they would say, like, you know, “Now we have to be cognizant that there can be like, families of difference”… but then they would go right back into it. So, it would be like “mom and dad.”

This participant, who had graduated within the previous 5 years, noted, “those assumptions rendered my existence invisible.” Similarly, participants remarked on the absence of LGBTQ+ content in health professions curricula, and too often what was present reinforced stereotypes. Many people reported their professional education programs having had a day devoted to LGBTQ+ health, with excessive focus on sexually transmitted infections.

In their professions, and in work contexts, participants were often advised not to disclose LGBTQ+ identities. One participant said she was warned by an older LGBTQ+ colleague, “Be careful who you tell.” Another was warned “to be more quiet about it”: “just saying like you better not talk about that, so-and-so staff member is really uncomfortable with that and may treat you differently.” This was echoed by another participant: “I actually had one of the preceptors [clinical educators] tell me—I said something about my partner and the preceptor actually said to me, ‘Ah, it's best not to mention that here.”' A participant who had a teaching role said it was routine for LGBTQ+ students to be warned not to share “anything about their personal identity” yet noticed this was never raised with cis-heterosexual students. As one participant commented, “Personally I don't really care, but it's just an odd comment to have staff take me aside and suggest that I should be less openly gay.”

Reflecting on heteronormativity in his profession, one participant questioned “if there's actually room for people that are more diverse.” Within his professional culture, he observed that “diversity isn't valued or welcome and that there's a certain mold that they kind of want, and they want everyone to be almost the same as much as possible. So, it does feel a bit unwelcoming.”

## Responding to heteronormativity

Pressure toward heteronormative conformity—assimilation—contributes to a lack of LGBTQ+ visibility in the health professions. As one participant commented, “In my medical school, there was over 200 students, and there was probably a handful of us that did identify as gay, but it wasn't particularly visible.” Others noted that even when there were other LGBTQ+ staff in their workplaces, those people rarely brought partners to workplace events, or talked about their personal lives: “They are pretty, um, pretty quiet about it.” While this cautiousness, guardedness is a response to heteronormativity, it simultaneously contributes to it, reinforcing a “spiral of silence” (Pasek et al., 2017, p. 401). Participants noted a particular dearth of LGBTQ+ visibility in the upper echelons of professions and workplaces, in senior administrative or leadership positions.

Making LGBTQ+ identities evident is not a single event; it entails a continuum from complete disclosure to complete non-disclosure (Stenger and Roulet, 2018). Most of our participants engaged in selective disclosure. Some had been more “out” before entering their professions, then “chose to be more and more closeted” as they progressed in their fields. As one participant said, “Students that are not out don't tend to come out during their [professional education]. They're afraid of what might happen. So there's still an abiding fear within even the younger generations.” Early years in practice were marked with considerable energy devoted to deciding whether, when and how to disclose at work, performing careful risk assessments: “A lot of thought in disclosure, and more so, I guess, at the beginning, less so now… In the beginning, it was, I did find it stressful.”

There was a general sense that disclosing LGBTQ+ identity was inappropriate in professional contexts: “It was a professional environment in the sense that it just never came up. I don't know how to say it. It just never came up. Like, with my colleagues, it was always about work. None of my personal stuff.” This was particularly strong regarding disclosing to clients; as one participant commented, “it's a professional boundary, right? I think for most people your first ‘go to' is going to be not disclosing too much.” Yet people had to actively decide how to respond to the heteronormative assumptions of coworkers and patients. As Stenger and Roulet found in their study of auditors, LGBTQ+ professionals engaged in “shamming, distance, and normification” (Stenger and Roulet, 2018, p. 267). In other words, passing (Goffman, 1963), distancing from others, and covering—striving to render queerness less objectionable to avoid stigma (Yoshino, 2006). Our participants did the same.

Passing can entail avoiding disclosure, but can also mean outright misdirection. As one participant said about responding to patient questions,

There would be many times that I'd just say, “Oh, I'm not married” or that sort of thing. I wouldn't give away anything more than that. And sometimes, I wouldn't even say anything. I would just kind of smile and nod and deflect.

Sometimes—depending on their assessment of the situation, as well as their own energy to engage—people actively misrepresented their LGBTQ+ identities, as described by a gay man:

[Patients] would say “Are you in a relationship?” and I would say “Yes,” and they would then say, “Oh, what does your girlfriend do?” and I would just be like, “Oh, she's an engineer.” Just to avoid the conversation, honestly, because in some instances… in a busy day, when I don't want to have an uncomfortable interaction, it felt easier to just lie.

One participant described his response as “declining to elaborate:” “I even sometimes find myself still kind of—not lying, but maybe sometimes still hiding, or you know just declining to disclose or elaborate.”

The process of selective disclosure relies on constantly assessing situations, calculating risk and benefit, plus the potential for disrupting normative expectations. As one lesbian health professional described, “Patients who are like, ‘Oh, do you have a boyfriend?' sometimes I'll say ‘No, I have a girlfriend,' and other times I don't, and it's a judgement that I make. Sometimes, you have to err on the side of caution.” As one participant argued, it is important to avoid “potentially opening yourself up to a whole world of hurt.”

One way of avoiding disclosures was detachment (Stenger and Roulet, 2018). One participant commented, “[I] found myself kind of avoiding talking about my personal life in a variety of situations,” including with patients and coworkers. Another had ceased engaging with colleagues socially, growing tired of dodging questions and comments that assumed heterosexuality: “So, I tend to avoid social situations as best as I can.” With clients, one participant stated that she always maintained a certain distance: “I definitely don't share much with my clients, just surface things.”

Yet, some people were concerned that distancing to avoid disclosure or heterosexist microaggressions could also hinder their ability to build rapport with patients:

You build a rapport with them over time. But, it's sort of like, I would always shut that door very, very quickly. And yeah, I feel like it doesn't allow for as natural an exchange as, say I had a husband… It seems almost easier to build rapport with clients when you're of that normative kind of status.

This participant “shut the door” on conversations about families and relationships, fearing for her safety if she disclosed LGBTQ+ identity; yet she worried keeping things superficial harmed her therapeutic work.

At the same time some participants found disclosing LGBTQ+ identities could also harm rapport, causing patients/clients to distance: “I've never had an experience where they were overtly homophobic. It's more people would stop opening up to me, or they would become suddenly very awkward and standoffish, and my rapport with them changed.” As another participant described, disclosure often disrupted connection: “It's probably only when you experience these things is when you notice it, but the pause or the facial expression is different…” In heteronormative work contexts, casual chatter about LGBTQ+ lives could hinder connection, but so could avoiding casual conversations. This is a distinct challenge in the health professions, where “therapeutic use of self” is part of establishing rapport.

Beyond not discussing LGBTQ+ identity, people put effort into impression management, disclosing they were LGBTQ+, but attempting not to look “too butch” or too “flamboyant:” “I would not buy bright colors for clothing. I would make sure that I sat with my legs crossed in a more male-identified manner... I would never let my hand rest down so that my wrist would fold.” This reflects what Yoshino calls “covering” and Stenger and Roulet (2018) call “normification:” “the strategy by which stigmatized individuals disclose some elements of their stigmatized identity while trying to present themselves as ordinary people” (Stenger and Roulet, 2018, p. 268). The prevalent deployment of heterosexist stereotypes meant participants could choose—to some extent—the degree to which they would embrace or counter expectations of LGBTQ+ embodiment and self-presentation (Einarsdóttir et al., 2016). The heteronormative assumption that sex, gender identity, gender expression, and sexual orientation fall neatly in line, gave participants some control over others' perceptions of them through managing their gender expression.

In the work context of the health professions, while some participants wished they had been less fearful of risk earlier in their careers, others reluctantly said heteronormative assimilation is an important strategy: “To kind of fit in with the way everyone else is.” This was identified as particularly important for trainees and those early in their careers.

I hate to say it because I don't think this is the best, but I think good advice might be to actually be more quiet… Sometimes being different can actually work quite against you… It's just not a culture that wants to promote diversity… If the people in power don't like that, then they can kind of um, maybe make you in a position where you are more likely to be ostracized.

Notably, within the health professions power hierarchies are multi-directional. LGBTQ+ health professionals may be constrained by the professional boundaries expected when providers are seen as inherently holding power relative to patients/clients, but also by workplace power structures intra-professionally and inter-professionally.

## Discussion

In Canada LGBTQ+ people have made gains regarding human rights protections which prevent or penalize the most flagrant instances of heterosexism and discrimination. Yet, while overt hostility may be decreasing, within the health professions heterosexist microaggressions appear to remain common (Eliason et al., 2018; Walker et al., 2022). Such incidents are difficult to prove definitively, let alone challenge. Our participants faced microaggressions from patients/clients as well as colleagues and managers, conveying a sense of inhospitability within their professions.

Perhaps even more significant than experiences of individual microaggressions, a culture of pervasive heteronormativity constituted LGBTQ+ health professionals as outsiders, as Other. Heteronormativity is institutionalized, built into everyday “business as usual.” As DePalma and Atkinson note, heteronormativity is supported in institutions “not only through what is said, but through silences, inferences and assumptions” (DePalma and Atkinson, 2010, p. 1,671). In our study, casual conversations with colleagues and with patients/clients—the everyday conversations through which workplace relationships and therapeutic rapport are built—were laden with potential pitfalls, sudden moments when health professionals needed to decide in an instant whether and how to disclose LGBTQ+ identity, while uncertain about the impact of disclosure. Consequently, some participants chose to remain distant from both colleagues and patients/clients, keeping connections superficial—which has its own costs. Heteronormativity was institutionalized in curricula, in admonitions about staying “closeted,” and in assumptions that professionalism is incompatible with open embodiment of LGBTQ+ social identities.

In the context of the health professions, rife with pervasive heteronormativity, both disclosure and non-disclosure of LGBTQ+ identities carry risk (Jackson, 2000; Risdon et al., 2000; Röndahl, 2011; Beagan et al., 2012; Robertson, 2017; Eliason et al., 2018; Gabrani and Pal, 2019; Murphy, 2019; Toman, 2019; Turban, 2019), resulting in what many participants described as pervasive invisibility of LGBTQ+ people, particularly higher in the power structures. Decisions about LGBTQ+ disclosure are affected by the extent of hierarchical power relations in a work setting (Vaccaro and Koob, 2019; Follmer et al., 2020). The health professions can be characterized as power-laden work contexts wherein trainees and junior professionals spend considerable time subject to high stakes assessments by powerful others (Martimianakis et al., 2009; Jenkins et al., 2021). As has been noted in other institutional contexts, even decisions not to disclose take untold energy and work, requiring “a carefully constructed system of strategic silences, half-truths and direct lies that … demand a great deal of attention and planning” (DePalma and Atkinson, 2010, p. 1,671).

Within health care workspaces, notions of professionalism may be mobilized against LGBTQ+ people to compel conformity with heteronormative expectations (see Mizzi, 2013). It is “unprofessional” to confront a patient/client who makes heterosexist comments or insults. Professionals are expected to be selfless, driven by altruism (Sibbald and Beagan, 2022). It is “unprofessional” to disclose LGBTQ+ identity because that disclosure is equated with talking about sex, which violates (hetero)professional boundaries (DePalma and Atkinson, 2010; Mizzi, 2013; Davies and Neustifter, 2021). When professionalism casts “proper” identities as devoid of sexuality, “sex and sexuality become too scandalous to mention within the rigid confines of a professional work circumstance,” leaving LGBTQ+ professionals outside the bounds of (hetero)professionalism by their very existence (Mizzi, 2013, p. 1,608).

Not surprisingly, many of our participants opted not to disclose LGBTQ+ identities, particularly with patients/clients. While this may well be a strategic move in heteronormative environments (Stenger and Roulet, 2018), nonetheless it contributes to queer erasure, rendering LGBTQ+ health professionals invisible. Opting to pass or assimilate, particularly in more risky work environments, perpetuates a “spiral of silence” (Pasek et al., 2017, p. 401), wherein LGBTQ+ learners and novices entering the professions discern that it is unsafe to be fully themselves in the health professions (Murphy, 2019). At the same time, the circulation of narrow discursive constructions of queerness means that LGBTQ+ health professionals who do disclose, or who are unable to conceal their sexual/gender identities, may face insistence that they engage in command performances of queerness that fit viewers' perceptions of the “right kind of queer.” The flip side of LGBTQ+ invisibility is hypervisibility, simultaneously casting the person as deviant, other, and reducing them to always/only ever their LGBTQ+ subjectivity (Einarsdóttir et al., 2016; Calvard et al., 2020; Davies and Neustifter, 2021).

### Queering the health professions

To queer, as verb, is to challenge what is considered normative, troubling it, creating disruptions, opening up spaces of possibility (Richards et al., 2017). To quote the late Canadian songwriter Leonard Cohen, “There is a crack, a crack in everything, That's how the light gets in” (Cohen, 1992). There are now numerous approaches advocated for responding to microaggressions at work, both as the person targeted and as a bystander who wants to act as an ally (see Sue et al., 2019). Too often these position the target of a microaggression as responsible for finding ways to defuse the situation and if possible, educate the perpetrator (Bullock et al., 2021). They emphasize staying open and curious, focusing on specific observations and employing “I statements,” avoiding judgement and expressing feelings, with an ultimate goal of “mutual understanding” (Torres et al., 2019, p. 870). For example, the “GRIT Framework for Addressing Microaggressions” asks those who experience microaggressions in health professional contexts to Gather themselves, Restate the comment, Inquire without judgement to gain clarification and Talk about the impact on self (Warner et al., 2020). Such frameworks put an exceptional burden on those who have just experienced something painful, threatening and/or diminishing to show “grit” and respond, well, professionally—without emotion.

From an extensive review of the literature, Derald Wing Sue (a key proponent of microaggression theory) and colleagues have identified four primary strategies in what they call microinterventions: make the aggression visible, disarm it, educate the offender and seek external support (Sue et al., 2019). They point out that while insufficient, responding to microaggressions can help shift workplace or professional cultures—though responses should take into account context, including power relations. In the medical education context, Bullock and colleagues place responsibility squarely on the shoulders of supervisors, suggesting they work closely with trainees facing microaggressions (Bullock et al., 2021). Anticipating microaggressions, supervisors should “pre-brief” with trainees, to identify preferred responses, then respond in the moment (always), followed by a debrief and possible formal action. Their model has promise. Others have suggested professionals might signal through imagery and language use that they are open to LGBTQ+ disclosures and willing to act as allies (Turban, 2019). Individual mentorship—and even more importantly, institutionally organized mentorship programs—for LGBTQ+ trainees and junior colleagues may also be helpful (Turban, 2019; Nair and Good, 2021; St John and Goulet, 2022; Westafer et al., 2022).

To return to Cohen's lyrics, how do we make “cracks” in heteroprofessionalism to “let the light in?” Our analysis suggests a need to counter the heteronormativity that pervades the health professions, through institutional, structural and cultural change such that heterosexism is no longer normative. This will require transformation in professional cultures and institutional environments—and perhaps most importantly in *status quo* notions of “professionalism.” As Davies and Neufstifter have argued, “normative ideas of professionalism encourage [workers] to not bring gender and sexual diversity or their lived queer and trans experiences actively into their [work]” (Davies and Neustifter, 2021, p. 3). Heteroprofessionalism (Mizzi, 2013) operates as a regulating force that needs disrupting.

The health professions have changed over decades, today reflecting much greater sociocultural diversity, yet concepts of professionalism have undermined that expansion, demanding conformity to monocultural norms and expectations. “Consensual discourses,” like the discourse of (hetero)professionalism, “may obscure and silence the expression of dissenting voices” (Turcotte and Holmes, 2021, p. 16). Transformation requires making space for dissidence in professional cultures (Davies and Neustifter, 2021; Turcotte and Holmes, 2021). It takes courage to disrupt, to resist, yet “our collective task is to interrogate and challenge the political, managerial and professional processes that regulate” (Turcotte and Holmes, 2021, p. 3). Acting individually carries risk; transforming heteroprofessionalism requires “collective enactment of disobedience” (Turcotte and Holmes, 2021, p. 6), radically disrupting the *status quo*.

## Limitations

This study was limited by conducting only single interviews with participants on a complex topic. This was a choice made to minimize participant burden, yet likely curtailed the depth of reflection possible. Secondly, including multiple professions in the sample allowed us to identify common patterns across fields, and particularly focus on professionalism as a regulating force, yet it also occluded attention to profession-specific details that may very well matter. A heterogenous sample may also hinder thematic saturation, the notion of “information redundancy,” though we certainly began to hear common narratives as the interviews progressed. Arguably, saturation is never reached in critical interpretive research, as the analysis spirals ever deeper, delving into unanticipated layers of meaning and interpretation (Braun and Clarke, 2021). Finally, the fact that almost all of our participants identified as cis-gender means analysis of the distinct experiences of transgender and gender diverse professionals remains under-analyzed. Similarly, the fact that our participants were almost exclusively white and able-bodied hindered analysis of the ways LGBTQ+ identities intersect with other marginalized social identities to shape experiences of belonging and marginality in the health professions.

## Conclusion

Despite advances in formal protections for LGBTQ+ people at work, health professionals may still face heteronormative environments that foster and are bolstered by heterosexist microaggressions. Those may be particularly challenging to address when they come from patients/clients, and particularly risky to address when they come from powerful professional others. Heteronormative assumptions convey subtle yet persistent messages of marginality, requiring LGBTQ+ health professionals to constantly navigate a tightrope between disclosure and non-disclosure, balancing personal safety against assimilation and LGBTQ+ invisibility. Often the ramifications of that navigation are uncertain, with any choice leading to possible harm. The precarity of this ongoing balancing act is predicated on heteroprofessionalism, the mobilization of “professional” as concept to undermine the credibility and validity of LGBTQ+ professionals, regulating their identity expressions and jeopardizing their ability to bring all of themselves to their work. While moves toward countering heterosexist microaggressions may help, particularly in the short-term, a more thoroughgoing transformation of heteroprofessionalism demands dissent and disruption to the very notion of professional.

## Data availability statement

Research ethics approval stipulated that data confidentiality would be maintained by the research team. Requests to access the datasets should be directed to ethics@dal.ca.

## Ethics statement

The studies involving human participants were reviewed and approved by Dalhousie University Health Sciences Research Ethics Board. Written informed consent was provided when interviews were conducted in person. When they were conducted by telephone, the written consent material was emailed to potential participants, then reviewed orally, with consent recorded before commencing the interview.

## Author contributions

All authors listed have made a substantial, direct, and intellectual contribution to the work and approved it for publication.
